# Mortality and illicit drug dependence among hemodialysis patients in the United States: a retrospective cohort analysis

**DOI:** 10.1186/s12882-016-0271-1

**Published:** 2016-06-08

**Authors:** Vanessa Grubbs, Eric Vittighoff, Barbara Grimes, Kirsten L. Johansen

**Affiliations:** Division of Nephrology, University of California, San Francisco, USA; Division of Nephrology, San Francisco General Hospital, 1001 Potrero Avenue, Building 100, Room 342, San Francisco, CA 94110 USA; Department of Epidemiology and Biostatistics, University of California, San Francisco, USA; Division of Nephrology, San Francisco Veterans Administration Medical Center, San Francisco, USA

**Keywords:** Illicit drug use, Hemodialysis, Mortality

## Abstract

**Background:**

Illicit drug use is common and known to cause and exacerbate a wide spectrum of kidney disease, often leading to end-stage renal disease (ESRD), but little is known about its prevalence or associated mortality among incident hemodialysis patients.

**Methods:**

This study is a retrospective cohort analysis using data obtained from the United States Renal Data System. We assembled a cohort of 511,821 incident hemodialysis patients age 20 years and older who initiated hemodialysis between January 1, 2006 and December 31, 2010. Illicit drug dependence was defined by comorbidity on the ESRD Medical Evidence Report (Form CMS-2728). We performed survival analysis to examine the association of drug dependence with overall mortality and mortality due to diagnoses that can be associated with intravenous drug use (drug-sensitive diagnoses) in the first year after initiating hemodialysis.

**Results:**

Drug dependence was recorded for 1.5 % (*n* = 7,461). Drug dependence was independently associated with a 1.3-fold and 2.5-fold higher hazard of overall mortality and mortality due to a potentially drug-sensitive diagnosis [adjusted hazard ratio (AHR) 1.34 (1.27–1.41) and 2.54 (2.05–3.14), *p* < 0.001, respectively]. This association varied significantly by age (p_interaction_ < 0.001), with a 9-fold higher hazard of mortality due to a potentially drug-sensitive diagnosis among the youngest patients with drug dependence [AHR 9.21 (5.15–16.44), *p* < 0.001].

**Conclusion:**

Illicit drug dependence is a burden within the ESRD program and is strongly associated with premature mortality, particularly among younger patients. Targeted intervention is needed to help reduce this burden.

**Electronic supplementary material:**

The online version of this article (doi:10.1186/s12882-016-0271-1) contains supplementary material, which is available to authorized users.

## Background

Since the passage of the Medicare End-Stage Renal Disease (ESRD) Program entitlement in 1972, dialysis has been transformed from a scarce resource available only to the young and otherwise healthy to an essentially unlimited therapy [1]. In-center hemodialysis facility availability has kept pace with increasing numbers of incident ESRD patients [2]. There have also been improvements in dialysis care since its inception, and mortality has declined by 28 % among hemodialysis patients over the last two decades. Nevertheless, little more than half are alive three years after ESRD onset [3].

**Table 1:** Patient characteristics by drug dependence, *N* = 511,821

Characteristic, *n* (% column)	No drug dependence (*N* = 504,360, 98.5 %)	Drug dependence (*N* = 7,461, 1.5 %)
Age group, years		
< 45	56,969 (11.3)	2,427 (32.5)
45–64	118,758 (37.4)	4,655 (62.4)
65–74	121,737 (24.1)	289 (3.9)
> 74	136,896 (27.1)	90 (1.2)
Male gender	283,718 (56.2)	5,286 (70.9)
Race		
White	331,019 (65.6)	2,703 (36.2)
Black	145,126 (28.8)	4,594 (61.6)
Asian	21,946 (4.3)	44 (0.6)
Other	6,269 (1.2)	120 (1.6)
Hispanic ethnicity	71,346 (14.1)	769 (10.3)
Primary cause ESRD		
Diabetes	232,612 (46.1)	1,893 (25.4)
Hypertension	145,548 (28.9)	2,568 (34.4)
Glomerulonephropathy	29,422 (5.8)	637 (8.5)
Other/Unknown	96,778 (19.2)	2,363 (31.7)
Vascular access at initiation		
AVF	70,741 (14.1)	605 (8.2)
AVG	17,344 (3.5)	227 (3.1)
Catheter with maturing AVF	78,717 (15.8)	1,048 (14.1)
Catheter with maturing AVG	12,296 (2.5)	226 (3.0)
Catheter only	320,720 (64.2)	5,304 (71.6)
No pre-dialysis nephrology care	161,201 (32.0)	4,073 (54.6)
Medicaid insurance	128,990 (25.6)	3,731 (50.0)

**Table 2:** Hazard of one-year mortality overall or due to drug-sensitive diagnosis,* by drug dependence

Model	No drug dependence	Drug dependence	
		Overall Mortality	Mortality due to potentially drug-sensitive diagnosis
	HR (95 % CI)	HR (95 % CI)	HR (95 % CI)
Adjusted for age, gender, race, ethnicity	1.0 (reference)	1.58 (1.50–1.67)	2.84 (2.30–3.50)
Adjusted for above + primary cause of ESRD	1.0 (reference)	1.52 (1.44–1.60)	2.80 (2.26–3.45)
Adjusted for above + pre-dialysis nephrology care and type of vascular access	1.0 (reference)	1.39 (1.32–1.47)	2.58 (2.09–3.19)
Adjusted for above + Medicaid insurance	1.0 (reference)	1.34 (1.27–1.41)	2.54 (2.04–3.14)

*p < 0.001 for all estimates

Although illicit drug use is common and known to cause and exacerbate a wide spectrum of kidney disease often leading to ESRD, little is known about its prevalence or associated mortality among incident hemodialysis patients [4–6]. A better understanding of the burden of illicit drug dependence among patients with ESRD could help identify important targets for improving the overall morbidity and mortality of the ESRD population. We examined the prevalence of and mortality associated with illicit drug dependence among incident hemodialysis patients in the United States using a national registry.

## Methods

### Study sample

Using data obtained from the United States Renal Data System (USRDS), we assembled a cohort of 511,821 incident hemodialysis patients age 20 years and older who initiated hemodialysis between January 1, 2006 and December 31, 2010. This study is deemed exempt from Institutional Review Board approval because it is a secondary analysis of de-identified data.

### Primary outcomes

Our primary outcomes were mortality, overall and due to diagnoses that can be associated with intravenous drug use (illicit drug-sensitive diagnoses) in the first year after initiation of dialysis. Patients were considered to have potentially drug-related mortality if any of the first three causes of death was attributed to valvular disease, septicemia secondary to internal vascular access, septicemia secondary to catheter, cardiac infection (endocarditis), or drug overdose from street drugs (Additional file 1).

### Primary predictor

Our primary predictor was illicit drug dependence, currently or within the last 10 years, as defined on the ESRD Medical Evidence Report (Form CMS-2728).

### Covariates

All covariates were defined using the Medical Evidence Report. Covariates included age, gender, race/ethnicity, primary cause of ESRD, Medicaid insurance, vascular access at dialysis initiation, and nephrology care prior to dialysis initiation. Age was categorized as 20–44, 45–64, 65–74, 75+ years. Race was categorized as white, black, Asian, or other; we also adjusted for an indicator of Hispanic ethnicity. Primary diagnosis of ESRD was categorized as diabetes, hypertension, glomerulonephropathy, or other/unknown. Vascular access at dialysis initiation was defined as ateriovenous fistula (AVF), ateriovenous graft (AVG), catheter with maturing AVF, catheter with maturing AVG, and catheter only. Duration of nephrology care prior to dialysis initiation was categorized as none, less than 6 months, 6–12 months, more than 12 months, or unknown. Finally, we considered Medicaid insurance as a proxy for socioeconomic status. Individuals were categorized as having Medicaid insurance if Medicaid was noted as the medical coverage at the time of dialysis initiation.

### Statistical analysis

Patient characteristics were examined by drug dependence using chi-square tests. We then used Cox proportional hazards models to examine the association of drug dependence with mortality overall and mortality due to potentially drug-sensitive diagnoses in the first year after initiating hemodialysis. To help understand confounding of drug dependence, we sequentially added primary cause of ESRD, pre-dialysis care and type of vascular access, and finally Medicaid insurance to a base model adjusting for age, gender, race, and Hispanic ethnicity. Finally, we examined the adjusted associations of drug dependence with mortality stratified by age at dialysis initiation and tested for interaction. Of note, we did not adjust for laboratory measures such as serum phosphate level or dialysis adequacy because these data are not included in the USRDS dataset. Regardless, while such measures may serve as markers of poor adherence and thus predict overall mortality, we view poor adherence as a potential mediator of the association between drug dependence and mortality, not a confounder. We used Shoenfeld residuals to assess the proportional hazards assumption, and estimated the association with drug dependence by quarter to examine the impact of the violations we found. All analyses were performed with Stata v. 14.0 (StataCorp, College Station, TX).

## Results

Patient characteristics are shown in Table 1. Among the 511,821 incident hemodialysis patients aged 20 years and older from January 1, 2006 to December 31, 2010, drug dependence was recorded for 1.5 % (*n* = 7,461). The prevalence of drug dependence decreased over time from peak prevalence of 1.56 % in 2006 to 1.4 % in 2010 (p_trend_ < 0.001) and was highest among those aged 45–64 years, males, and blacks. Half of patients with drug dependence had Medicaid insurance and slightly more than half had no nephrology care prior to initiating hemodialysis (54.6 %).

**Fig. 1:**
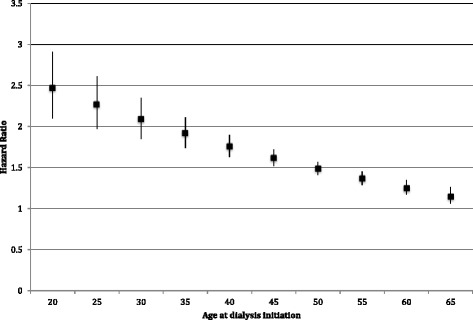
Adjusted hazard ratios for the association of drug dependence with first-year overall mortality, by age at dialysis initiation

Approximately one-quarter (22.9 %) of the population died within the study period (n = 117,326). No cause of death was recorded for 23.3 % (*n* = 27,342) of deaths in the first year. Missing cause of death was slightly more common among those with drug dependence than those without (27.7 % vs. 23.3 %, *p* < 0.001). Drug dependence was associated with a 1.6-fold higher hazard of overall mortality after adjusting for demographics [partially adjusted hazard ratio (HR) 1.58 (1.50–1.67), *p* < 0.001] (Table 2). The association was partially attenuated after adjusting for other covariates, but remained statistically significant [fully adjusted HR 1.34 (1.27–1.41), *p* < 0.001]. This association varied significantly by age (p_interaction_ < 0.001), with the highest hazards of overall mortality due among the youngest patients but remaining statistically significantly higher for older patients (Fig. 1).

**Fig. 2:**
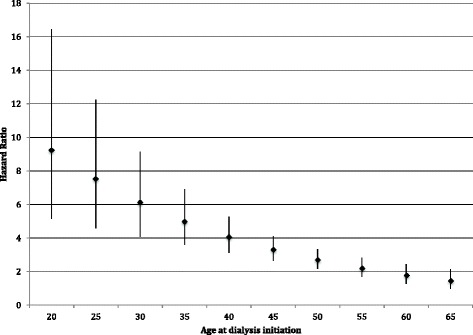
Adjusted hazard ratios for the association of drug dependence with first-year mortality due to drug-sensitive diagnosis, by age at dialysis initiation

Drug dependence was associated with a 2.8-fold higher hazard of mortality from potentially illicit drug-sensitive diagnoses after adjusting for demographics [partially adjusted HR 2.84, 95 % CI (2.30–3.50), *p* < 0.001] (Table 2). This association was also partially attenuated after further adjustment, but remained statistically significant [fully adjusted HR 2.54 (2.05–3.14), *p* < 0.001]. As with overall mortality, this association varied significantly by age (p_interaction_ < 0.001), but with much higher hazard ratios. The highest hazard ratios for the association of drug dependence and mortality due to potentially drug-sensitive diagnosis were among the youngest patients and remained statistically significantly higher until age 65 (Fig. 2).

In our analyses of sensitivity to the proportional hazards assumption, we found somewhat weaker but still statistically significant associations of drug dependence with both mortality endpoints in the first quarter, compared to the remainder of the first year.

## Discussion

Within this national registry of incident hemodialysis patients, we found that illicit drug dependence was associated with significantly higher rates of overall mortality and mortality due to diagnoses that can be associated with illicit drug use, particularly among younger hemodialysis patients. Given that over 40,000 people in the United States died of drug-induced causes in 2010—exceeding other preventable deaths such as those from firearms or alcohol, [7] our findings are not surprising. However, this is the first study to our knowledge to quantify the prevalence of illicit drug dependence and associated mortality in this population. Among patients under the age of 40, drug dependence was independently associated with a 4-to 9-fold increased hazard of dying of a potentially drug-sensitive diagnosis. In contrast, drug dependence among older patients does not appear to signal increased risk, possibly because it reflects more remote exposure or survival bias.

While almost all dialysis patients under age 40 are expected to survive for at least one year, [8] the sharply higher mortality rates we observed among younger drug-dependent patients, in particular deaths due to potentially drug-related causes, suggest that these patients comprise a high-risk subgroup requiring targeted interventions. In our study, 4 % (approximately 1,600) of patients under age 40 requiring renal replacement each year have illicit drug dependence. Attention to reducing morbidity and mortality in this population is urgently needed.

Because dialysis resources are not scarce in the United States, it is the general consensus that any patient who clinically requires renal replacement therapy and agrees to dialysis should have it. Accordingly, nearly all drug-dependent patients in need of renal replacement therapy receive hemodialysis. Since illicit drug use is known to cause and exacerbate renal disease, and non-adherence to the medical regimen is common in this population, active drug use is considered a contraindication to renal transplantation—the form of renal replacement therapy affording the best survival and quality of life [9–11]. Homelessness and inadequate housing are common in this population, and often render peritoneal dialysis a non-viable option for renal replacement therapy, due to the high risk of peritonitis and lack of storage space for dialysis materials. Furthermore, injection drug use damages blood vessels, which often destroys potential sites for permanent vascular access. Therefore the only attainable vascular access is often a dialysis catheter, which is not only prone to high rates of infection, but may also be used by the injection drug user for intravenous drug use, with its inherent risk of overdose and additional risk of infection. As a result, drug dependent patients in need of renal replacement therapy are relegated to care that places them at increased risk of morbidity and mortality attributable to ESRD, overdose, and infection. Further, since both transplant and peritoneal dialysis are also considerably less costly than hemodialysis, drug dependent patients are also given the most expensive care—thus disproportionately contributing to the estimated $11 billion annual healthcare cost attributed to illicit drug dependence [12].

Ongoing drug use and personal behaviors common among dialysis patients with illicit drug dependence, such as non-adherence to a standard dialysis prescription, also contribute to high risk of morbidity and mortality [13]. Yet there is currently no requirement that drug dependent patients undergo treatment for their addictions. Drug rehabilitation programs are readily accessible and considered effective treatment for drug addiction. While rates of participation in drug rehabilitation among the dialysis population are unknown, participation in the general population is very low [14]. In a 2013 survey, only 13.4 % of persons age 12 or older with illicit drug dependence or abuse received treatment for their illicit drug use in the past year [15]. Further, 80.9 % did not perceive a need for treatment for their illicit drug use [15]. However involuntary or coerced treatment, e.g. through drug treatment court, has been demonstrated to be effective means of achieving sobriety and decreasing future recidivism [16]. For societal and individual benefit, the illicit drug dependent population with ESRD should be strongly counseled to actively participate in existing but sorely underutilized drug treatment programs at the time of dialysis initiation and as a part of ongoing dialysis care. Social workers are available in dialysis facilities to address patients’ psychosocial issues, but the scope is often limited to dialysis-related concerns. At the very least, dialysis providers should inform drug dependent patients of the substantial morbidity and mortality associated with their addiction, but awareness and harm reduction programs alone have not been shown to fully curtail continued risky behavior [17, 18].

This study is not without limitations. We identified illicit drug dependence using Form 2728. Ascertainment of comorbid conditions using this form has been shown to have excellent specificity but lack sensitivity [19]. Moreover, the under-ascertainment of drug dependence may be non-random, with minorities, young patients, and the poor more likely to be asked about illicit drug use than their white, older, and non-poor counterparts. The resulting under-ascertainment of drug dependence among older patients and whites, who are both at increased mortality risk compared to younger patients and minorities, would likely bias our estimates of the effects of drug dependence towards the null. Further, illicit drug dependence on Form 2728 is defined to include use within last 10 years, so that mortality associated with current use is likely higher than observed in our study. Similarly, Form 2728 does not ascertain route of illicit drug use. Some illicit drug use may only be in pill or inhaled forms, and would not entail increased risk of our potentially drug-sensitive diagnoses, with the exception of overdose. Thus the estimated 2.5-fold higher risk of death from those diagnoses may understate the actual increase among intravenous drug users. In addition, cause of death was missing for approximately one-quarter of patients who died in the first year, potentially resulting in biases either towards or away from the null. However, such bias is unlikely to account for the strong associations we found. Finally, we were not able to examine hospitalization and procedure burden associated with drug dependence because these data are collected only on Medicare beneficiaries, whereas nearly two-thirds (63 %) of those with drug dependence were not Medicare-eligible by 90 days after dialysis initiation.

## Conclusion

Despite these limitations, this study provides evidence of the burden of illicit drug dependence within the ESRD program and its strong association with mortality, particularly among younger patients. Targeted intervention is needed to help reduce this burden.

## Abbreviations

AVF, ateriovenous fistula; AVG, ateriovenous graft; ESRD, end-stage renal disease; USRDS, United States renal data system

## Additional file

Additional file 1: Drug-sensitive diagnosis codes. Contains cause of death diagnoses considered potentially drug-related. (DOCX 41 kb)
