# Supplementary material for: Mortality and illicit drug dependence among hemodialysis patients in the United States: a retrospective cohort analysis
Source: BMC Nephrol. 2016 Jun 8;17:56. doi: 10.1186/s12882-016-0271-1 (PMC4898454; doi:10.1186/s12882-016-0271-1)
Supplement: Additional file 1: — Drug-sensitive diagnosis codes. Contains cause of death diagnoses considered potentially drug-related. (DOCX 41 kb) [file 12882_2016_271_MOESM1_ESM.docx]

Additional file 1. Drug-sensitive diagnosis codes

| Code | Diagnosis |
| --- | --- |
| 30 | Valvular heart disease |
| 33 | Septicemia due to internal vascular access |
| 34 | Septicemia due to vascular access catheter |
| 61 | Cardiac infection (endocarditis) |
| 93 | Drug overdose (street drugs) |
